# Organisational implementation climate in implementing internet-based cognitive behaviour therapy for depression

**DOI:** 10.1186/s12913-022-08041-y

**Published:** 2022-05-31

**Authors:** Christiaan Vis, Annet Kleiboer, Mayke Mol, Claus Duedal Pedersen, Tracy Finch, Jan Smit, Heleen Riper, on behalf of the MasterMind Consortium, Olatz Albaina, Marco Cavallo, Els Dozeman, Claus Duedal Pedersen, David Ebert, Anne Etzelmüller, Erik van der Eycken, Ane Fullaondo, Andrea Gabilondo, Ana González Pinto, Begoña Gutiérrez, Annet Kleiboer, Elisabeth Kohls, Esteban de Manuel, Kim Mathiasen, Mayke Mol, Joana Mora, Luisa Peleteiro-Pensado, Joaquín Ponte, Kevin Power, Ander Retolaza, Heleen Riper, Ylenia Sacco, Anneke van Schaik, Modesto Sierra Callau, Mette Maria Skjøth, Jan Smit, Melita Sogomonjan, Maria Tajes-Alonso, Jon Txarramendieta, Christiaan Vis, Chris Wright, Enrico Zanalda

**Affiliations:** 1 Department of Clinical, Neuro-, & Developmental Psychology, Faculty of Behavioural and Movement Sciences, VU Amsterdam, Van der Boechorststraat 7, Amsterdam, 1081 BT The Netherlands; 2 Amsterdam Public Health Research Institute – Mental Health, Amsterdam, Netherlands; 3 World Health Organization (WHO) Collaborating Centre for Research and Dissemination of Psychological Interventions, Amsterdam, Netherlands; 4 Dept. of Psychiatry, Amsterdam University Medical Centre (VUmc), Amsterdam, The Netherlands; 5 Sentinel Unit, Sundhed.dk, Odense, København Denmark; 6 Department of Nursing, Midwifery & Health, Northumbria University, Northumbria, United Kingdom

**Keywords:** Organisational Implementation Climate, Organisational Context, Internet-based Cognitive Behavioural Therapy, Acceptance, Implementers, Service deliverers

## Abstract

**Background:**

Internet-based Cognitive Behaviour Therapy (iCBT) for depression have been implemented in routine care across Europe in varying ways, at various scales and with varying success. This study aimed to advance our understanding of organisational implementation climate from the perspectives of implementers and mental health service deliverers.

**Methods:**

Qualitative and quantitative methods were combined to study the concept of organisational implementation climate in mental health care settings. Based on concept mapping, a qualitative workshop with implementers was used to conceptualise organisational implementation climate for optimizing iCBT use in routine practice. Service deliverers involved in the provision of iCBT were invited to participate in an explorative cross-sectional survey assessing levels of satisfaction and usability of iCBT, and organisational implementation climate in implementing iCBT. The two methods were applied independently to study viewpoints of implementers as well as service deliverers. Corresponding to the explorative nature of the study, inductive reasoning was applied to identify patterns and develop a reasonable explanation of the observations made. Correlative associations between satisfaction, usability and implementation climate were explored.

**Results:**

Sixteen implementers representing fourteen service delivery organisations across Europe participated in the workshop. The top-three characteristics of a supportive organisational implementation climate included: (1) clear roles and skills of implementers, (2) feasible implementation targets, and (3) a dedicated implementation team. The top-three tools for creating a supportive implementation climate included: (1) feedback on job performance, (2) progress monitoring in achieving implementation targets, and (3) guidelines for assessing the impact of iCBT. The survey (*n*=111) indicated that service providers generally regarded their organisational implementation climate as supportive in implementing iCBT services. Organisational implementation climate was weakly associated with perceived usability and moderately with satisfaction with iCBT services.

**Conclusions:**

Organisational implementation climate is a relevant factor to implementers and service deliverers in implementing iCBT in routine care. It is not only an inherent characteristic of the context in which implementation takes place, it can also be shaped to improve implementation of iCBT services. Future research should further theorise organisational implementation climate and empirically validate the measurement instruments such as used in this study.

**Supplementary Information:**

The online version contains supplementary material available at 10.1186/s12913-022-08041-y.

## Background

Depressive disorders are amongst the most prevalent mental health conditions around the world [1]. Internet-based Cognitive Behavioral Therapy (iCBT) can increase reach and accessibility of mental health services [2] with comparable efficacy to face-to-face CBT [3–5]. Moreover, iCBT services in general are found to be appropriate and acceptable strategies in treating depression [6–8]. Consequently, various initiatives emerged across the globe to implement iCBT services in routine care [9, 10]. Implementation here, is to be understood as a deliberate and planned process of integrating and embedding whereby an innovation becomes a normal part of daily routine [11–13].

**Fig. 1:**
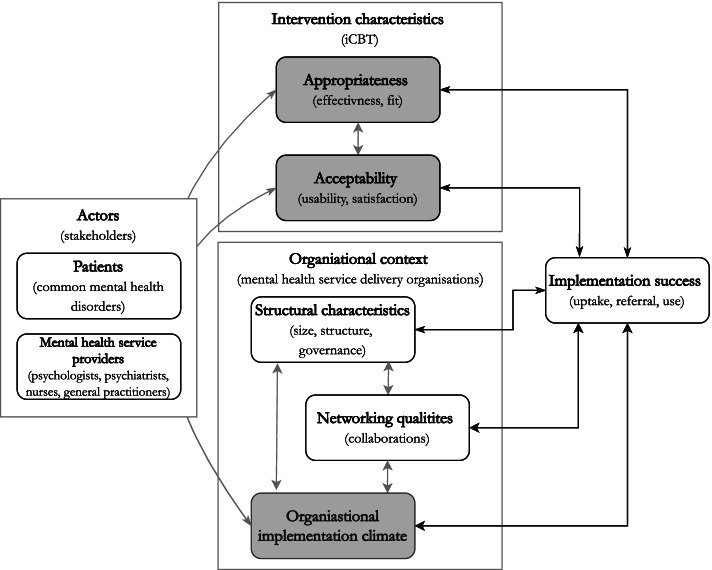
Conceptual model of implementation success, intervention characteristics and wider organisational context. The relation between organisational implementation climate and acceptability of iCBT services that were implemented, was the subject of this explorative study

Clinical effectiveness, perceived appropriateness and acceptability by mental health service deliverers are known to be important determinants of successful implementation of iCBT services in routine care [14–17]. Appropriateness refers to the suitability of iCBT in treating depressive disorders and acceptability concerns the perception of users including patients and service deliverers that the iCBT service is palatable or satisfactory in its use [18]. Besides individual level factors related to iCBT, also the context in which it is implemented on group level can hamper or facilitate implementation efforts [19]. These contextual factors can operate on the level of the health care system, (e.g. rules for reimbursement, certification, and staff accreditation), as well as on organisational level (e.g. procedures, structures, social characteristics, human and financial resources) [16]. One could argue that barriers on the level of organisational context might be more sensitive to change whereas system level barriers are often outside the influence of implementers. Furthermore, the organisational context in which the service delivery and implementation takes place, is of particular relevance as it forms the ‘ecosystem’ in which patients and service deliverers act and interact to create health and healthcare [20, 21]. Figure 1 provides a schematic simplification of a possible model of implementation success indicating potential relations between appropriateness and acceptability of the intervention, the wider organisational context, and main actors involved in delivery and uptake of iCBT services (i.e. patients and service deliverers).

Organisational context as defined in the Consolidated Framework for Implementation Research (CFIR), includes structural characteristics such as age, size, and governance structure of the organisation, networking qualities which refer to formal and informal communications within and beyond the organisation, and aspects of the organisational implementation climate by which implementation processes are facilitated or inhibited [19]. Of particular interest is the concept of organisational implementation climate which can be defined as the shared meaning staff members attach to organisational events, practices and procedures they experience and the behaviours they see being rewarded, supported, and expected in implementing new practices [22–25]. Organisational implementation climates are possibly relevant to investigate as they are known to shape staff members’ attitudes towards adopting and implementing new interventions into daily practice [23, 24, 26] and professionals perceptions and attitudes are of particular interest in successfully implementing new interventions [27, 28]. Organisational implementation climate is a conglomerate concept that includes staff members’ shared understanding of and experiences with organisations’ formal and informal policies and practices (e.g. training) related to implementing new interventions [29]. Through discussion and collaboration, and in the context of these organisational policies and practices, staff members develop a collective sense of what is expected from them, how this can be achieved, and what possible consequences it might cause for them.

Applied to the context of adult mental health care, organisational implementation climate can be characterised in various ways. For example, one characteristic concerns service deliverers’ commitment and loyalty to the organisation and its’ goals. Commitment and loyalty to the organisation can be considered to be part of a broader concept about individuals’ identification and relationship with that organisation and may affect the willingness to implement and use novel interventions such as iCBT services [19, 30]. Another defining characteristic is employees’ perceptions of the levels of support, recognition and appreciation by their organisations for implementing new interventions as it can incentivise individuals to adapt or apply a certain behaviour facilitating implementation practices. [19, 23, 31]. For mental health service deliverers, examples of such incentives can include salary raises, a promotion to a supervisory role, gratifications, conference visits, and increased stature, respect and trust by granting more autonomy in treating their patients. Another characteristic of organisational implementation climate includes staff members’ confidence in their own ability to change their practice and use new interventions such as iCBT in treating their patients. This notion of self-efficacy is a significant component in most individual behaviour change theories [32, 33]. Furthermore, professionals’ shared perception of the importance of implementing new interventions could be a relevant aspect shaping organisational implementation climates [19, 23, 31]. Similarly, the availability of qualified staff, number and adequacy of resources such as funds, training, and time available to implement and use the new services might be relevant factors characterising organisational implementation climates as they can enable or hinder actual enactment of implementation efforts [11, 19].

In the European MasterMind project, unguided, guided and blended iCBT interventions for adults suffering from mild, moderate or severe depressive disorder were implemented in fourteen European regions [34]. The aim of this project was to scale up the use of iCBT services across Europe and by doing so, conduct a summative evaluation of barriers and facilitators in implementing these services in a variety of mental health settings [34]. It also provided the possibility to advance our understanding of the nature and value of organisational implementation climate in implementing such services in routine mental health care from two perspectives. From the perspective of implementers, i.e. staff members tasked with implementing the iCBT services, we sought to qualitatively identify (a) the characteristics of and (b) practical tools for creating an organisational implementation climate conducive to improving implementation success. In addition, we quantitatively explored and described mental health service deliverers’ perspective to gain an initial understanding of the relevance of the concept of organisational implementation climate and whether measures of satisfaction, usability are empirically associated with organisational implementation climate in samples of mental health service deliverers.

## Methods

### Study setting

The study ran from September 2015 until January 2017 [34]. Table 1 provides an overview of the iCBT services and the organisations involved in the Mastermind project. All implemented iCBT services were based on the main therapeutic principles of Cognitive Behaviour Therapy (CBT), covering sessions of psychoeducation, behavioural activation, and cognitive restructuring. Two regions implemented services that were designed as standalone self-help interventions by which only technical assistance was available to patients. Four regions implemented iCBT services that included a secure asynchronous messaging system by which therapists could offer coaching to the patients using the iCBT service. Two regions implemented iCBT services with a blended treatment protocol by which therapeutic sessions delivered face-to-face or by videoconferencing are combined with online sessions and asynchronous therapeutic guidance. The participating organisations were divided into two implementation waves. Wave-1 organisations were more experienced in providing iCBT services and at the time, could be considered as early adopters due to their involvement in previous research and implementation projects of iCBT services. Wave-2 organisations had limited experience with iCBT services for depressive disorders in routine care and benefitted from sharing knowledge, (parts of) interventions, and lessons learned with wave-1 organisations in developing and implementing iCBT services.

**Table 1:** Demographic characteristics of the organisations and iCBT services implemented in the MasterMind project

Wave	Org. ID.	Region, country	iCBT service	Guidance modality^1^	Referral pathways^2^	Referrals^3^ *n*	Reach^4^ %	Org. size^5^	Funding^6^
1	1	Scotland, UK	Beating the Blues	Self-help	GP, SP	5,724	5.30	M	Public
1	2	Southern Denmark, DK	NoDep	Guided	S	259	0.72	S	Public
1	3	Amsterdam area, NL	MindDistrict, MoodBuster	Blended	GP, SP	355	3.31	L	Insured
1	4	Hospital group and online provider, DE	Depression Online, Relapse prevention, GET.ON Mood enhancer, Get.On Sleep	Blended	S, O	1,405	0.26	L	Insured
1	5	Tromsø area, NO	MoodGym	Self-help	GP	191	5.46	M	Public
2	6	Basque Country, SP	Super@tuDepression	Guided	GP, SP	216	0.55	L	Public
2	7	Wales, UK	Beating the Blues	Self-help	GP	355	3.34	L	Public
2	8	Aragon, SP	Super@tuDepression	Guided	SP	129	3.00	M	Other
2	9	Badalona, SP	Super@tuDepression	Guided	GP	253	1.01	L	Other
2	10	Galicia, SP	Super@tuDepression	Guided	GP	110	0.11	L	Public
2	11	Piemonte, IT	iFightDepression	Guided	S, GP, SP	161	0.75	L	Other
2	12	Veneto, IT	iFightDepression	Guided	SP	150	0.17	S	Other
2	13	Anatolia, TR	Top Sende	Guided	S	120	1.42	S	Other
2	14	Harju, EE	iFightDepression	Guided	S	56	1.60	S	Insured

1 Guidance modality refers to a categorisation of the online and face-to-face human interaction in the iCBT service. S: self-help by which none or only technical and administrative support is provided. G: therapeutic guidance provided by a therapist online through asynchronous messaging. B: blended in which sessions in face-to-face or videoconferencing format are integrated with online sessions in one treatment protocol

2 Main patient referral pathway to the iCBT service. GP: via General Practitioner offices; SP: via mental health specialist referral; S: self-referral; O: other, e.g. via health insurers

3 Referrals concerns the total number of patients deemed eligible for the iCBT service and received an account to access the treatment. Eligibility was determined following local clinical guidelines and was based on clinical judgement and/or using a structured validated clinical questionnaire (e.g. PHQ-9)

4 Reach is the proportion of eligible individuals in a given (estimated) catchment area and those actually involved in the service

5 Indicator of the size of the mental healthcare organisation involved in the implementation based on an estimate of the annual revenues and number of employees. L: large organization (revenues > 50 mln. Eur., full time equivalent (FTE) staff positions > 500). M: medium-large organisation (revenues 10-50 mln. Eur., FTE < 500). S: small organisation (revenues < 2 mln. Eur., FTE < 200)

6 Indicator of the source of funding source of the iCBT service. Insured: service use is reimbursed by private health insurances. Public: service is reimbursed by the public health care system. Other: project-based, out of pocket expenses, other sources or a combination of these

### Methods

Two methods were combined to study the concept of organisational implementation climate in mental health care settings. A qualitative conceptualisation workshop was used to gather data from implementers about (1) characteristics of and (2) practical tools for shaping a supportive organisational implementation climate. Cross sectional survey data was collected to quantitatively describe organisational implementation climate and explore correlations with scores of perceived iCBT service satisfaction and usability amongst mental health service deliverers. The two methods were applied independently of each other to elucidate viewpoints from the two different target groups (implementers and service deliverers). Corresponding to the explorative nature of the study, inductive reasoning was applied to identify patterns in the data and develop a logical explanation of the observations made.

### Conceptualisation workshop

A concept mapping approach [35] was used to identify, cluster and rank ideas for two separate themes: 1) characteristics of an organisational implementation climate specifically focused at fostering successful implementation of iCBT in routine practice, and 2) practical tools implementers use to create and facilitate such supportive organisational implementation climates. Implementers involved in coordinating or executing the implementation of the iCBT services were eligible to participate in the workshop. Two implementers of each region participating in the MasterMind study were invited to participate in the workshop (see Table 2). Following the concept mapping approach, the workshop was structured into four separate steps for both themes to ensure a participatory conceptualisation process [35]:

1. Generate ideas: all participants individually wrote down as much as possible initial ideas concerning the afore mentioned two themes in fifteen minutes. This ‘silent groups’ format preserves individuality but introduces a possible social facilitation effect from the presence of others.
2. Merge ideas: in group setting, the ideas generated were recorded in rotation, one idea per person on an electronic screen. The rotation procedure removes some of the anonymity of a ‘talk in any order’ group while at the same time producing a list of ideas that are recorded without authorship.
3. Refine ideas: continuing in the group setting, the ideas from steps one and two were clarified, discussed, combined, or refined as the group saw fit. One idea was discussed at a time and individuals were asked for reasons of agreement or disagreement and constructive suggestions for improvement. Combining and refining was done based on their perceived similarity and the revised ideas were recorded in a new list visible for the whole group.
4. Ranking: as a final step, each participant independently and silently rated the revised ideas in terms of its importance or usefulness to the theme. Ranking was achieved by averaging the individual votes for each theme.

**Table 2:** Demographics of the conceptualisation workshop participants

Variable	Pooled	Wave 1	Wave 2
Sample, *n*	16	8	8
Age in years, *M* (SD)	39.3 (10.9)	41.5 (12)	37 (10.1)
Min. – max.	26-61	29-61	26-59
Gender, *n*			
Female	8	5	3
Profession, *n*			
MH professional^1^	7	4	3
Service dev., proj. mgr.^2^	4	1	3
Director, leadership	3	1	2
Consultant, advisor	2	2	0
Managing role, *n*			
Yes	6	3	3
Field experience, *n*			
< 3 years	3	0	3
3 – 5 years	4	3	1
6 – 10 years	5	3	2
> 10 years	4	2	2
Experience with iCBT, *n*			
Yes	7	6	1

1 MH professional means mental health professionals such as psychiatrist, psychologist, mental health nurse, etc

2 Service dev., proj. mgr. means roles of service developer or project manager

The concept mapping workshop was facilitated by members of the central MasterMind project evaluation team (CV, MM and AK) and designed to last maximal four hours divided into two main parts. The workshop was conducted face-to-face during a MasterMind consortium meeting in Turin, Italy on 13 October 2016.

### Cross-sectional survey

The survey focussed on service deliverers’ perception of the organisational implementation climate they experienced in the organisation they worked for and their satisfaction with and usability of iCBT services. These were applied descriptively.

An explorative questionnaire was developed to obtain a preliminary quantitative assessment organisational implementation culture. We defined organisational implementation climate as the shared meaning service deliverers attach to organizational events, practices and procedures they experience and the behaviours they see being rewarded, supported, and expected in implementing new interventions. Starting from this definition and existing literature, the central research team (CV, AK, MM, HR) deductively developed an initial pool of questions. This initial list was improved and corroborated in two review rounds by members of the MasterMind consortium during the start-up phase of the MasterMind project, i.e., prior to the qualitative workshop. The resulting 12 questions related to commitment, loyalty, support, recognition, appreciation, self-efficacy, relative priority, resources, and implementation strategies. Commitment was measured with two questions assessing individual participants perception of their own and of their supervisors’ commitment to the organisations’ goals. Loyalty to the organisation was measured by one question addressing respondents’ own allegiance to the organisation they work for. The extent to which respondents perceive to be incentivised by their organisation was assessed with three questions asking the extent to which respondents felt being supported, recognised, and appreciated when implementing and using iCBT in daily service provision work. Aligned with Bandura’s work [36], self-efficacy was measured with two questions addressing respondents’ confidence in their own abilities and enthusiasm in implementing and using iCBT service in practice. The perceived availability of resources for implementing iCBT in practice was measured by two questions concerning the availability of qualified staff to provide the iCBT services, and the availability of other resources such as time, training, computers, etc. The extent to which respondents regarded the implementation as deliberate and planned was measured by one question asking about the existence of an implementation strategy for implementing the iCBT service. All questions were rated using a 5-point Likert answering scale ranging from ‘1. strongly disagree’ to ‘5. strongly agree’. Service deliverers could rate a question as ‘not applicable’ when the question was perceived to be irrelevant to their situation or organisation.

Satisfaction with the iCBT services was measured with the short version of the Client Satisfaction Questionnaire (CSQ-3) using a 4-point scale with three items [37]. It has good psychometric properties, and it has been tested in numerous studies on diverse samples of patients and professionals [38, 39]. Following the questionnaire instructions, scale scores were calculated by summing item ratings. Higher ratings indicate higher levels of satisfaction.

Usability was measured with the System Usability Scale (SUS) using a 5-point Likert scale to rate ten items [40, 41]. It has good psychometric properties and is tested in numerous studies including samples of mental health professionals [40, 42, 43]. For calculating the SUS scale item’s score contribution ranged from 0 to 4. Negative worded items were converted to adhere to the same range order. Score contributions of each item was summed and multiplied with 2.5 resulting in a scale of 0 - 100 [41]. Higher scale scores are indicative of higher levels of usability.

Mental health service deliverers involved in the provision or referral of patients to the iCBT services, such as licenced psychotherapists, psychiatrists, mental health nurses, and general practitioners, were eligible to be included in the cross-sectional survey. Depending on local circumstances in the participating MasterMind regions, various recruitment strategies were applied, including open, electronic mass mailings, and targeted individual mailings. Starting from January 2015 for wave-1 and October 2015 for wave-2 sites, service deliverers’ demographics were collected the moment they enrolled into the MasterMind project. Organisational implementation climate (OIC), satisfaction (CSQ), and usability (SUS) were surveyed in both wave 1 and wave 2 sites at the end of the study in December 2016. The survey was administered online and in local language (Danish, Dutch, English, Estonian, German, Italian, Norwegian, and Spanish) using existing translations. The survey was translated by external translators and checked by the local investigators when no translations were available. Data was uploaded to the central MasterMind database using a standardised codebook. The survey is included in Additional file 2.

### Statistical analyses

Survey data was cleaned using descriptive statistics assessing distributions, centrality, outliers and missing values. We did not impute data. Three of the fourteen organisations were exposed to considerable participant drop-out due to staff turnover during the data collection period leading to severe case nonresponse and therefore excluded from the analysis. Overall there were 120 cases in the data set of which nine were removed due to severe item nonresponse. That is, 111 cases responded to at least one item on satisfaction, usability and organisational implementation climate questionnaires (44:57 wave 1 to wave 2 ratio). In total, 80 cases completed all questions of all questionnaires. 103 completed all SUS items, 108 all CSQ items, and 89 responded to all OIC questions. Total OIC scores were calculated by taking the sum of scores for each question. We assumed that higher scores are indicative of a stronger organisational implementation climate. Scale scores for SUS and CSQ were calculated following the respective prescribed scoring systems. That is, for CSQ we used summed item rating scores. For SUS the summed item ratings were converted to a 0-100 scale using the curved grading scale by Sauro et al. [44], i.e. a score of 68 was considered as the centre of the scale and thus as ‘average’ in comparison to norm data. Cronbach’s alpha (α) was calculated as a measure for internal consistency of SUS, CSQ and OIC in this particular sample. We considered 0.70 < α < 0.90 as indicative of a good internal consistency [45]. 95% Confidence intervals (95%-CI) around are α were reported to prevent over interpretation. We expected considerable heterogeneity amongst the participating implementers and service deliverers within implementation regions due to the design of the MasterMind project (e.g. wave-1, wave-2 representing experience in delivering iCBT) and geographic diversity and subsequent health systems the service delivery organisations operated in. To gain a descriptive understanding of this variety, differences in demographics and scores due to experiences with implementing and delivering iCBT services between Wave-1 and 2 implementers and service deliverers were analysed using the Wilcoxon rank sum test with continuity correction. The non-parametric Wilcoxon rank sum test was used because of the 4 and 5-point scales used for which the data cannot be assumed to follow a normal distribution. A 95% confidence interval was used. We calculated Spearman's rank-order correlation coefficient (*r*_s_) to explore the strength and direction of correlation between OIC questions and SUS, and between OIC and CSQ respectively. We applied the following strength indicators for the correlations: 0 ≤ *r*_s_ < 0.3 is weak, 0.3 ≤ *r*_s_ < 0.5 is moderate, and *r*_s_ ≥ 0.5 is strong [46]. Data cleaning and statistical analysis was carried out in R [47] using RStudio [48] using packages psych [49], ggplot2 [50] and sjPlot [51].

## Results

### Conceptualisation workshop

Table 2 provides an overview of the demographic characteristics of the participants of the conceptualisation workshop. Implementers were on average of middle age (M* =* 39.3 years, SD = 10.9) and had a clinical mental health background (*n* = 7 with 5-10 years of experience in the field of mental health (*n* = 5). Seven out of sixteen implementers had previous experience with iCBT services and six had a management role in the organisation.

#### Theme 1: characteristics of an organisational implementation climate fostering successful implementation of iCBT in routine practice.

A total of 55 items were generated for theme one identifying characteristics of a positive organisational implementation climate in the first individual silent brainstorming round. The items were merged, refined and conceptualised in 9 clusters in group discussions. The clusters were ranked by each participant individually. The results of the workshop including generated ideas, clusters and ranking outcomes are included in Additional file 1. The top-3 ranked clusters of characteristics of a supportive organisational implementation climate included: (1) clarity on role and skills of implementers, (2) feasibility of implementation targets, and (3) instigating a dedicated implementation team.

#### Theme 2: practical tools to create and facilitate a positive organisational implementation climate.

The second theme addressed practical tools implementers can use to create and facilitate an organisational implementation climate that improves implementation outcomes. For this theme 29 items were generated by the workshop participants individually and in silence. In a structured group discussion (second and third step of the conceptualisation workshop), the items were refined and merged in 10 clusters. In the last step, participants ranked the clusters individually. The ideas, clusters and ranking outcomes are included in Additional file 1. The top-3 ranked clusters of characteristics of practical tools can be used to build a supportive organisational implementation climate included 1) providing regular and structured job performance feedback in delivering iCBT, (2) structurally monitor use of iCBT and implementation progress, and (3) practical guidelines and methods for impact assessment of new interventions such as iCBT in this case.

### Cross-sectional survey: demographics, satisfaction, usability and organisational implementation climate

Table 3 presents the demographic data and two items regarding the perceived state of change and efficiency gains in delivering iCBT services. Most service deliverers were female (*n* = 80, 73%), psychologists (in training or licensed, *n* = 50, 45%) or general practitioners (GP, *n* = 31, 28%) with more than 10 years of experience in the field of mental health care. Most service deliverers across both waves had limited experience with delivering iCBT (*n* = 62, 58% used iCBT with patients less than 4 times). However, service deliverers in wave-1 had significantly more experienced in providing iCBT than wave-2 participants (W = 1739.5; 95%-CI = 0.00, 1.00; *p* = .01). Most service deliverers received iCBT specific training and the two groups did not differ in their response (W = 1601; 95%-CI = 0.00, 1.00; *p* = .24). When asked about their perceived state of change in using iCBT, a third (*n* = 33, 33%) indicated to perceive delivering iCBT services as a normal practice, and one third (*n* = 34, 34%) was trialling delivering the service. Significantly more wave-2 service deliverers were in the phase of gaining insight and trialling its use than wave-1 participants (W = 1618; 95%-CI = 0.00, 1.00; *p* = .01). The fact that wave-2 differed significantly from wave-1 service deliverers in their experience in iCBT delivery and their state of change aligns with what expected differences between organisations with more experience in implementing iCBT services (wave-1) and those with less experience (wave-2).

**Table 3:** Extended demographics of delivery staff, pooled and per implementation wave

Variable	Pooled	Wave 1	Wave 2
Sample, *n* (%)	111 (100)	48 (43)	63 (57)
Gender, *n* (%)			
Female	80 (73)	36 (77)	44 (70)
Profession, *n* (%)			
GP	31 (28)	0 (0)	31 (49)
Licenced psychologist	20 (18)	10 (21)	10 (16)
Psychologist in training	30 (27)	29 (62)	1 (2)
Psychiatrist	6 (5)	1 (2)	5 (8)
General mental health worker	6 (5)	1 (2)	5 (8)
Other	17 (15)	6 (13)	11 (17)
Experience in mental health care, *n* (%)			
< 3 years	18 (17)	7 (15)	11 (18)
3 – 5 years	18 (17)	12 (26)	6 (10)
6 – 10 years	23 (21)	15 (32)	8 (13)
> 10 years	49 (45)	13 (28)	36 (59)
Experience with iCBT, *n* (%)			
Provided a patient < 4 times iCBT	62 (58)	19 (42)	43 (69)
Provided a patient 5 – 10 times iCBT	11 (10)	8 (18)	3 (5)
Provided a patient 11 – 15 times iCBT	8 (8)	6 (13)	3 (5)
Provided a patient 16 – 20 times iCBT	4 (4)	0 (0)	4 (6)
Provided a patient > 20 times iCBT	21 (20)	12 (27)	9 (15)
Received iCBT training, *n* (%)			
Yes	82 (75)	38 (81)	44 (71)
If yes: type of iCBT training received, *n* (%)^1^			
Technical	34 (39)	6 (20)	28 (49)
Therapeutic	4 (5)	1 (3)	3 (5)
Both	47 (54)	23 (77)	24 (42)
Other	2 (2)	0 (0)	2 (4)
State of change in delivering iCBT, *n* (%)^2^			
Orienting	8 (8)	4 (8)	4 (8)
Gained some insight	22 (22)	8 (17)	14 (26)
Decided to change	4 (4)	2 (4)	2 (4)
Trialling usage	34 (34)	10 (21)	24 (45)
It is normal	33 (33)	24 (50)	9 (17)
Perceive an efficiency gain through delivering iCBT, *n* (%)^3^			
Strongly disagree	3 (3)	1 (2)	2 (4)
Disagree	13 (13)	8 (19)	5 (9)
Disagree nor agree	29 (29)	15 (35)	14 (25)
Agree	41 (41)	10 (23)	31 (54)
Strongly agree	14 (14)	9 (21)	5 (9)

1 Item-nonresponse: 21.6 % due to not all service deliverers received a training prior to filling out the demographics survey

2 Item-nonresponse: 9%

3 Item-nonresponse: 10%

#### Scores and item ratings

**Fig. 2:**
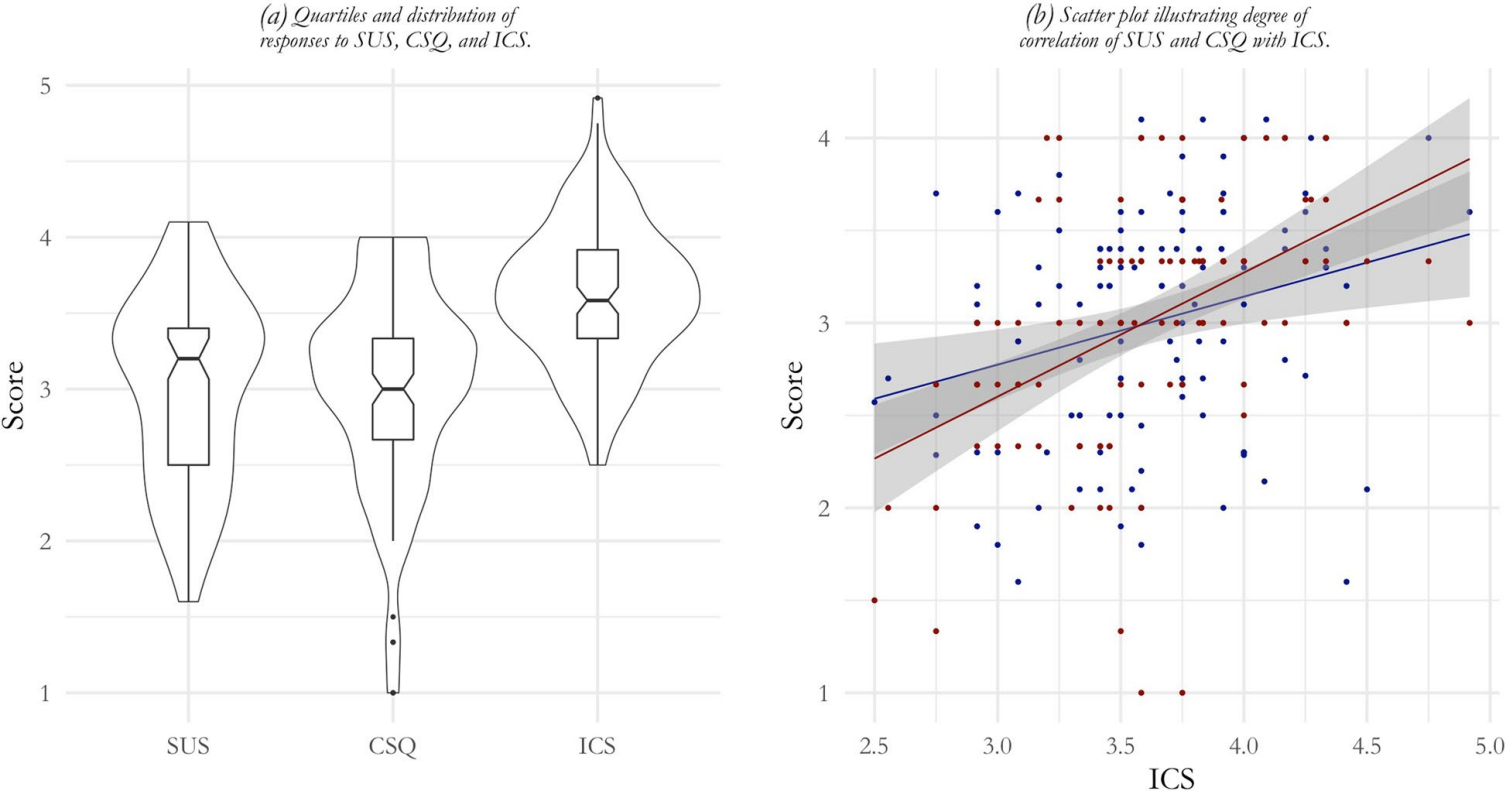
**a** Boxplot indicating the quartiles and response distribution of the SUS, CSQ and OIC questions. **b** Scatter plot indicating the distribution of item responses and illustrating degree of correlation of responses for SUS and CSQ items with OIC questions. Blue and red dots represent SUS and CSQ data points respectively. The blue and red lines represent the linear regression models between respectively SUS and OIC, and CSQ and OIC. The shaded area indicates the 95% confidence interval around the regression lines

Service deliverers regarded the usability of iCBT services as slightly below average (*M*_SUS_ = 63.76; SD = 15.53) and satisfactory (*M*_CSQ_ = 9.11; SD = 1.96). Similarly, organisational implementation climate was also rated slightly above neutral with a total mean score of 43.21 (SD = 5.62). Table 4 provides the statistics for each questionnaire. Detailed item scores are included in Additional file 2. All questionnaires had good internal consistency (α_SUS_ = 0.83, 95%-CI = 0.75, 0.9; α_CSQ_ = 0.82, 95%-CI = 0.73, 0.89; α_OIC_ = 0.76, 95%-CI = 0.64, 0.85). Wave-2 service deliverers scored significantly different on the SUS scale (W = 1919.5; 95%-CI = 7.50, 17.50; *p* < .05), but not on the CSQ (W = 1569.5; 95%-CI = 0.00, 1.00; *p* = .42) and the IOC questionnaire (W = 907.5; 95%-CI = -3.00, 2.00; *p* = .52). The Boxplot in Fig. 2a also indicates that service deliverers agree in their perceived usability (SUS) of iCBT services, and the organisational implementation climate (OIC) they operate in. As indicated in Fig. 2b, organisational implementation climate was weakly associated with variation in the system usability scale (*r*_s_ = 0.25; *p* = .03), and moderately correlated with the client satisfaction scale (*r*_s_ = .51; *p* ≤ .00).

**Table 4:** Item and questionnaire scores of perceived usability (SUS-10) and satisfaction (CSQ-3) with iCBT services and organisational implementation climate (OIC) by professionals at post study

Measure^1^ Wave	Item^2^					Scale^3^					
	*n*	Mean (SD)	Median	Min	Max	*n*	Mean (SD)	Median	Min	Max	Alpha^4^ (95%CI)
SUS-10	111	3.04 (0.29)	3.00	2.14	3.80	103	63.76 (15.53)	67.50	27.50	90.00	0.83 (0.75-0.90)
Wave 1	48	2.99 (0.20)	3.00	2.70	3.60	48	70.26 (10.82)	72.50	42.50	90.00	0.76 (0.65-0.85)
Wave 2	63	3.08 (0.34)	3.10	2.14	3.80	55	58.09 (16.84)	57.50	27.50	90.00	0.84 (0.77-0.90)
CSQ-3	111	3.02 (0.66)	3.00	1.00	4.00	108	9.11 (1.96)	9.00	3.00	12.00	0.82 (0.73-0.89)
Wave 1	48	3.13 (0.51)	3.00	2.00	4.00	48	9.40 (1.54)	9.00	6.00	12.00	0.65 (0.49-0.78)
Wave 2	63	2.93 (0.75)	3.00	1.00	4.00	60	8.88 (2.23)	9.00	3.00	12.00	0.89 (0.83-0.93)
OIC	111	3.62 (0.46)	3.58	2.50	4.92	89	43.21 (5.62)	43.00	30.00	59.00	0.76 (0.64-0.85)
Wave 1	48	3.57 (0.46)	3.54	2.75	4.92	47	42.96 (5.50)	43.00	33.00	59.00	0.76 (0.64-0.85)
Wave 2	63	3.66 (0.47)	3.70	2.50	4.75	42	43.50 (5.81)	43.00	30.00	54.00	0.77 (0.67-0.86)

1 SUS (10 items) applied a 5-point Likert scale with 1 = strongly disagree to 5 = strongly agree. Negative SUS items were rescored to align with positive worded items. CSQ (3 items) applied a 4-point scale with differing response options indicating agreement with statements. OIC (12 questions) applied a 5-point Likert scale with 1= strongly disagree to 5 = strongly agree

2 Item statistics using raw item ratings. All cases with more than one item rated were included

3 Scale statistics using summed item rating scores. For SUS-10, the summed item ratings were converted to a 0-100 scale following Brook (1996). Only complete cases were included

4 Standardised Cronbach’s alpha using a correlation matrix

## Discussion

In this study, a qualitative concept mapping workshop was combined with an exploratory cross-sectional survey to advance the understanding of organisational implementation climate in implementing iCBT services in mental health care settings. The aim was to obtain a qualitative understanding of how implementers characterise organisational implementation climate and substantiate this with a preliminary quantitative exploration amongst mental health service deliverers in an organisational context.

The main findings from the concept mapping workshop are aligned with Klein and Sorra’s integrative model of determinants of the effectiveness of organisational implementation [23]. In their model, implementation effectiveness is in part a function of the strength of an organisation’s climate for implementation which comprises a set of organisational policies and practices. According to this theory, different organisational policies and practices may be equifinal in their outcome, skills and motivation play an important role in achieving sustained use of the innovation as unskilled, unmotivated are unlikely to use the innovation at all [23]. This confirms what implementers ranked high in the workshop regarding roles, capabilities and skills of implementers, implementation targets, and the competences of the implementation team as a whole. Similarly, these findings are aligned with the Normalisation Process Theory (NPT) which takes a sociological perspective in theorizing the way people act and interact in integrating and embedding new ways of working in existing practices [52]. For example, the importance of skill sets in organising collective action, corresponds with the finding that for implementers to be effective, they need to have the position and role in the implementation work and team that fits their capabilities and skills. In addition, having realistic implementation time frames, and practical and feasible targets can influence how the new intervention is used in practice. This corresponds to NPT’s notion of interactional workability as a factor shaping collective action through operationalization of the innovation into practical ways of working that fit the local context. Furthermore, the finding that members of an implementation team should have a shared interest and beliefs in the implementation goals, corresponds to the theory’s notion of coherence referring to processes of individually and collectively determining the innovation’s practical meaning and utility.

Turning to the cross-sectional survey, mental health service deliverers were generally satisfied with iCBT (*M*_CSQ_ = 9.11, SD = 1.96) and regarded usability of the iCBT services as slightly below average (*M*_SUS_ = 63.76, SD = 15.53). These acceptability scores are slightly more positive than existing literature on clinicians’ perspectives toward delivering Internet-based psychotherapies. In a German survey comparing acceptance of web-based psychotherapy, it was found that clinicians scored around the summed midpoint of the scale (total score = 45.18, scale range = 16-80, *n* = 428) indicating a more neutral stance [53]. Another study found an overall a neutral stand point (*M* = 3.45, SD 0.72, 5-point Likert scale with 3 as neutral score, *n* = 95) on a survey designed to contrast perceived advantages and disadvantages of Internet-based therapies among Austrian psychotherapists [54]. A third study reported similar score patterns of perceptions of computer-based psychological treatments (*M* = -0.05, SD = 0.79, 5-point Likert scale with 0 as neutral score, *n* = 26) [55]. This difference in perceived acceptance of Internet-based psychotherapies might be explained by that the majority of the service deliverers (82%) involved in the MasterMind project received iCBT training prior to filling out the survey whereas 80% of the participants in the Schröder study indicated to have no or limited prior knowledge of Internet-based interventions. This might indicative of that the samples were drawn from different groups of mental health service deliverers and the possible difference between intended use by non-experienced professionals and actual use by trained professionals. In addition, the difference in findings might be due varying study designs applied. In our study we choose to use more generic instruments (SUS and CSQ) whereas in the other studies applied questionnaires that were specifically developed for the studies’ purposes.

Pending examination of the validity of the OIC questions, a third finding in this explorative study is that a stronger organisational implementation climate is (weak to moderate) associated with higher levels of satisfaction and usability of iCBT. Although causality is not proven by this study, this finding could lead to proposing that acceptability of iCBT services in terms of usability and satisfaction, might vary as a function of organisational implementation climate. That is, more supportive organisational implementation climates might enhance service deliverers’ acceptance of iCBT services. Although in this study the measures of usability and satisfaction are iCBT specific, this is aligned with an earlier finding concluding that organisational climate is associated with mental health service deliverers’ attitudes towards deciding to adopt evidence-based practices in general [24]. This American study amongst public sector professionals providing youth and family mental health services, showed that supportive organisational cultures for implementing evidence-based practices were associated with positive attitudes of participants towards those practices. Similarly, a weak organisational implementation climate was associated with higher levels of perceived discrepancies between current and new ways of working, most notably when there are unclarities and conflicts about roles and responsibilities. Authors concluded that clear specification of deliverers’ roles and actions can enhance implementation climates and subsequent contribute to implementation success. This aligns with findings from our conceptualisation workshop, where it was ranked as first characteristic of a strong organisational implementation climate conducive of improving implementation outcomes. This reasoning needs to be considered with care as the OIC has been developed pragmatically and requires further investigation of the validity, accuracy and reliability in assessing organisational implementation climates.

When viewed in combination, the qualitative findings from the workshop on the characteristics of a supportive organisational implementation climate conceptually align with the explorative survey used in this study. Despite the pragmatic approach, the questions related to commitment, attitudes, and resources conceptually seem to align to implementers’ notions of people and skills, the implementation team, availability of resources and attitudes. This makes sense because, for example, attitudes as referring to the perceived self-esteem in using a new intervention found in the workshop, directly corresponds to a survey item about confidence in ones’ own ability to implement. Similarly, the importance of resources supportive to the implementation work such as incentives, skilled people and champions, time, technology, technological support, and policies, qualitatively aligns to survey questions addressing availability of qualified staff, adequate resources, and implementation strategies. In that respect, the findings of the workshop combined with the survey suggests that organisational implementation climate is not only an inherent property of the context in which implementation activities take place, it can also be intentionally shaped to enhance impact of those activities.

### Strengths and limitations

This study contributed to an initial understanding of organisational implementation climate in mental health care settings from the viewpoint of implementers, and service deliverers who are required to deliver innovative iCBT services. By combing different viewpoints and methods in one study, a more comprehensive understanding of organisational implementation climate in relation to implementing iCBT services in mental health settings is provided.

However, the findings of this study should be interpreted with care for several reasons, including the inevitable heterogeneity in the settings in which the organisations implemented these iCBT services, and the representativeness of implementers and service deliverers in implementing and delivering the services. Service organisations not only varied in their position in the regional health care system (primary, secondary care), they also varied in their sources of funding for delivering mental health services (Table 1) driven by underlying regional and national policy contexts. Although in general, most mental health service organisations in Europe are transitioning towards deinstitutionalised care [56], the organizations participated in the MasterMind project likely had differing objectives in implementing the (self-selected) iCBT service. In relation to that, it must be noted that partaking in the MasterMind project and receive (complementary) European funding for implementing and evaluating iCBT services, might have impacted decision-making and enactment of implementation activities. Furthermore, the implementers at the organisations recruited the service deliverers for the survey which might have led to a biased sample of service deliverers who had an interest in innovation and international collaborations in the field of mental health.

Besides the heterogenetic settings, several methodological limitations need to be considered. The workshop was highly structured. Participants received instructions in advance of the meeting, a combination of pen-and-paper and digital recording methods were used, as well as individual silent idea generation and rankings and structured one-by-one group clarification discussions were used to prevent production blocking [57]. The workshop was held in English. Because only two participants were native English speakers, cognitive inertia might have been induced pursuing participants to the same line of thinking and potentially leading to fear of being judged and pressured to remain within the scope of existing options. Although the workshop was designed to include silent individual and group work, this pressure might have influenced the performance of the group in generating a rich variety of ideas during the first two steps and ranking of ideas later on. For the quantitative survey it must be noted that although the questions are aligned with the explorative and pragmatic nature of the study, the empirical validity of the findings represented by the OIC questions is unclear. Question generation and selection were based on a literature review and expert assessment of whether the questions made sense and were meaningful in a mental health setting. We have not performed an empirical psychometric assessment to validate the conceptual and psychometric properties of the questions and the latent constructs they might or might not represent.

### Future research

A notion of organisational implementation climate in implementing iCBT services in routine mental health care has been explored in this study. Open phenomenological research is required to further theorise the concept and mechanisms by which organisational implementation climates exerts change in implementing iCBT in mental health settings. In coherence with this theoretical work, research should focus on developing a reliable, valid, and practical questionnaire to quantify organisational implementation climates. Such questionnaire along with other data sources could then be used to empirically confirm the theoretical assumptions and improve our understanding of the complex interactions between the iCBT, implementers, service deliverers and the organisational context they operate in. In this respect, one important research question could be concerned with how and to what extent organisational implementation climates can be used as an active implementation strategy to effectively improve implementation outcomes.

## Conclusion

This study aimed to advance the understanding of the nature and value organisational implementation climate in implementing iCBT services in routine mental health care settings. The qualitative findings from the concept mapping workshop conceptually align with the quantitative approach applied in this study for measuring organisational implementation climate. This suggests that organisational implementation climate is not only an inherent characteristic of the context in which implementation takes place, it might also be shaped to improve the impact of those activities in implementing iCBT services in routine care settings. From the perspective of implementers, a supportive organisational implementation climate includes (1) clarity on skills and roles of implementers, (2) feasibility of implementation targets, and (3) instigating a dedicated implementation team. The top-three tools that can be used to create a supportive implementation climate include: (1) job performance feedback, (2) monitoring in progress in achieving implementation targets, and (3) providing guidelines and protocols for structured impact assessment. From the perspective of mental health service deliverers, the organisational implementation climates they operated in was perceived as supportive to implementing the iCBT services. Explorative analysis revealed that organisational implementation climate was weakly associated with usability and moderately with satisfaction scores. Considering the explorative nature of the current study, future research should theorise and improve the OIC into a valid and accurate instrument for assessing organisational implementation climate. Such empirically validated instrument can be used to design and test implementation interventions that are designed to enhance and use implementation climates for improving implementation outcomes.

## Supplementary Information

**Additional file 1. **

**Additional file 2.**

## Data Availability

To preserve anonymity of the participants of the qualitative parts of this study, the datasets used and/or analyzed during the current study are available from the corresponding author on request.

## Abbreviations

CFIR: Consolidated Framework for Implementation Research
CSQ: Client Satisfaction Questionnaire
GP: General Practitioner
iCBT: Internet-based Cognitive Behaviour Therapy
OIC: Organisational Implementation Climate
MasterMind: MAnagement of mental health diSorders Through advancEd technology and seRvices– telehealth for the MIND
NPT: Normalisation Process Theory
SUS: System Usability Scale
