# Supplementary material for: Organisational implementation climate in implementing internet-based cognitive behaviour therapy for depression
Source: BMC Health Serv Res. 2022 May 31;22:720. doi: 10.1186/s12913-022-08041-y (PMC9153170; doi:10.1186/s12913-022-08041-y)
Supplement: Supplementary file 1 — Additional file 1. [file 12913_2022_8041_MOESM1_ESM.docx]

The relevance of Organisational Implementation Climate in implementing internet-based Cognitive Behaviour Therapy for depression. An explorative cross-sectional study of implementers and service deliverers

Additional file 1: Results brainstorming workshop, ranked clusters and items

Christiaan Vis - p.d.c.vis@vu.nl

| Cluster | Ranking *M (SD,*  Min. – Max.) | Items mentioned in the silent brainstorm |
| --- | --- | --- |
| *Theme 1: Characteristics of an organisational implementation climate that is conducive to better implementation outcomes.* | | |
| People and skills | 6.5 (2.2, 2–9) | People involved in the implementation work should be in a position and have a role that suits their capabilities. Leadership skills in successfully implementing iCBT services. |
| Feasibility | 5.5 (2.6, 1-9) | Implementation goals or targets should be practically feasible. |
| Implementation team | 5.4 (2.4, 1-9) | Clarity on roles, responsibilities and managerial hierarchy; selection of voluntary team members who are willing and able; balanced experience and drive/ambition of the team; a strong shared interest and belief in the implementation goal; healthy competitiveness. |
| Available resources | 5.0 (2.5, 1-9) | Availability of facilities to adopt to new technologies, incentives to use iCBT, key persons in the implementation process, a legal framework related to the iCBT interventions, time and resources for learning and education of professionals, supporting national health policies, access to [technical] support, peer-pressure, iCBT experts/champions, and leadership assistance and support. |
| Alignment with strategic and procedural context | 4.7 (2.6, 1-9) | iCBT service provision should be aligned with the organisational goals; impact on bureaucracy should be clear as well as the patient needs. Open feedback should be possible about treatment outcomes, case load performance. |
| Transparency | 4.4 (2.9, 1-9) | Tailored acceptance through dialogues with positive and negative stakeholders highlighting different dimensions of the implementation problem; openness to critical voices; transparency about the objectives and aims of implementing the iCBT services; understanding the benefits and of the barriers to implementing iCBT; clearly defined expectations and outcome performance measures; all partners should have an idea of why they are here and what the added value for stakeholders is to using iCBT services. |
| Accessible information | 4.3 (2.4, 1-8) | Information about patient outcomes and satisfaction with the iCBT services should be accessible, understandable, and presented in appropriate manners; provision of best practices in using iCBT. |
| Stakeholders | 4.1 (2.5, 1-9) | Reasonable number and active involvement of all relevant stakeholders in the implementation; embedded implementation leader and use of external iCBT champions. |
| Attitudes | 3.8 (2.1, 1-7) | Belief and perseverance in pursuing implementation goals; self-esteem in delivering iCBT services and acceptance of failures; Flexibility in applying to real patients, and sympathy and understanding that things can go wrong; a learning environment; openness of clinical professionals in general and to technology and computers specifically; inclusiveness and taking care of ego's; a focussed implementation solution; promotion of collaboration in delivering iCBT; attitude of fluency, friendliness and ‘do what you preach’; shared understanding, believe in, and acceptance of the benefits of the iCBT service. |
| *Theme 2: Practical tools for creating and maintaining an organisational implementation climate that is conducive to better implementation outcomes.* | | |
| Refinements | 8.2 (2.3, 4-10) | Regular feedback about job performance, and service delivery outcomes to refine implementation strategies and activities. |
| Performance management | 6.8 (2.1, 3-10) | Follow-up on milestones, deliverables and use key performance indicators; regular check data collection status and quality. |
| Protocols | 6.3 (2.7, 2-10) | Develop and use medical guidelines and protocolise data collection procedures for evaluating iCBT service outcomes and implementation success. |
| Preparation | 5.6 (3.0, 1-9) | Conduct a situation analysis and needs assessment; reserve time to test and pilot the iCBT intervention and implementation activities before engaging in full blown implementation trajectories. |
| Training | 4.9 (1.8, 1-7) | Staff development and training; peer-coaching between implementers and clinicians involved in the implementation work; events organised for exchange experience; draw on previous experience via communication; cooperation through workshops; collaborative training. |
| Infrastructure | 4.8 (2.0, 2-8) | Use of multidisciplinary and participatory framework to involve key stakeholders; adequate, accessible, and reliable equipment and technical support; reliable data collection and analyses tools. |
| Encouraging resources | 4.4 (2.9, 1-10) | To encourage implementation team members explicitly by eg. celebrating successes, special attention, dinners, and thoughtful and inspiring emails. |
| Communication | 4.1 (2.7, 1-10) | A clear communication plan that identifies target audiences, the general meaning of the main messages and media to convey the message. Also enable personal communication with stakeholders through email and preferably telephone; open dialogue using meetings and discussions about strategic planning; basic communication and presentation skills of the implementers are required. |
| Motivation and involvement | 4.1 (2.7, 1-9) | Transparent and fair payment structures and providing (financial) incentives; involvement of the whole implementation team; conduct site visits; celebrate success. |
| Planning | 3.1 (2.7, 1-10) | Acceptance of those involved by having a clear decision-making process; include a clear and easy to understand project Gantt-chart with milestones and clear endpoints; plan meetings with stakeholders at the right time; list biggest challenges and involve patients generating solutions. |
