# Supplementary material for: Organisational implementation climate in implementing internet-based cognitive behaviour therapy for depression
Source: BMC Health Serv Res. 2022 May 31;22:720. doi: 10.1186/s12913-022-08041-y (PMC9153170; doi:10.1186/s12913-022-08041-y)
Supplement: Supplementary file 2 — Additional file 2. [file 12913_2022_8041_MOESM2_ESM.docx]

The relevance of Organisational Implementation Climate in implementing internet-based Cognitive Behaviour Therapy for depression. An explorative cross-sectional study of implementers and service deliverers

Additional file 2: Survey items and results

Christiaan Vis - p.d.c.vis@vu.nl

**Results surveys**

Bar graph of item scores per scale. Note that for CSQ a 4-point scale was applied lacking a neutral response category. SUS and ICS use a 5-point Likert scale. To increase readability of the graphs some words in the items are left out. This is indicated with brackets [..] and mostly concerned reference to a description of the iCBT service.


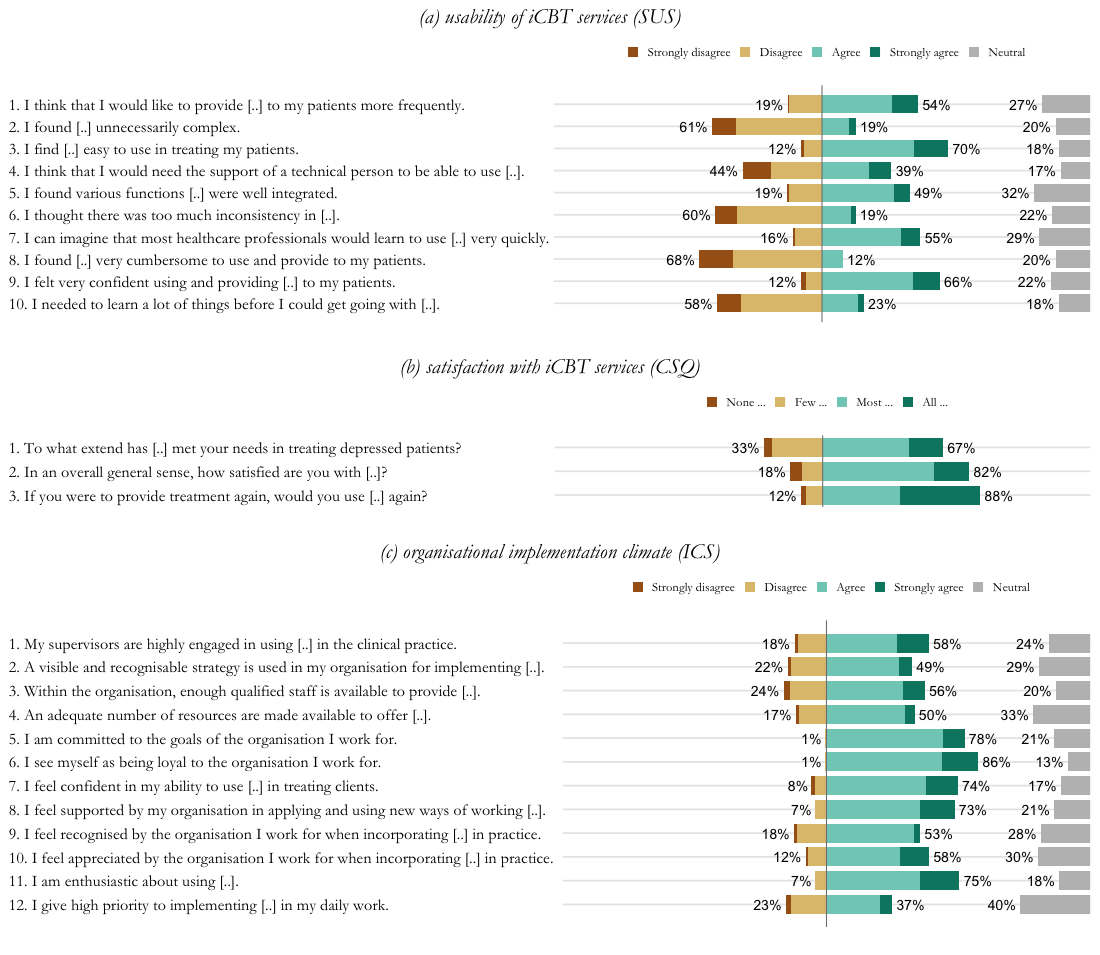


**Survey Items**

**System Usability Scale**

1. I think that I would like to provide the iCBT intervention to my clients more frequently.

1 = I strongly disagree

2 = I disagree

3 = I don´t disagree nor agree

4 = I agree

5 = I strongly agree

99 = Not answered

2. I found the iCBT intervention unnecessarily complex.

1 = I strongly disagree

2 = I disagree

3 = I don´t disagree nor agree

4 = I agree

5 = I strongly agree

99 = Not answered

3. I find the iCBT intervention easy to use in treating my clients.

1 = I strongly disagree

2 = I disagree

3 = I don´t disagree nor agree

4 = I agree

5 = I strongly agree

99 = Not answered

4. I think that I would need the support of a technical person to be able to use and provide the iCBT intervention to my clients.

1 = I strongly disagree

2 = I disagree

3 = I don´t disagree nor agree

4 = I agree

5 = I strongly agree

99 = Not answered

5. I found various functions in the iCBT intervention were well integrated.

1 = I strongly disagree

2 = I disagree

3 = I don´t disagree nor agree

4 = I agree

5 = I strongly agree

99 = Not answered

6. I thought there was too much inconsistency in the iCBT intervention.

1 = I strongly disagree

2 = I disagree

3 = I don´t disagree nor agree

4 = I agree

5 = I strongly agree

99 = Not answered

7. I can imagine that most healthcare professionals would learn to use and provide the iCBT intervention very quickly.

1 = I strongly disagree

2 = I disagree

3 = I don´t disagree nor agree

4 = I agree

5 = I strongly agree

99 = Not answered

8. I found the iCBT intervention very cumbersome to use and provide to my clients.

1 = I strongly disagree

2 = I disagree

3 = I don´t disagree nor agree

4 = I agree

5 = I strongly agree

99 = Not answered

9. I felt very confident using and providing the iCBT intervention to my clients.

1 = I strongly disagree

2 = I disagree

3 = I don´t disagree nor agree

4 = I agree

5 = I strongly agree

99 = Not answered

10. I needed to learn a lot of things before I could get going with using and providing the iCBT intervention to my clients.

1 = I strongly disagree

2 = I disagree

3 = I don´t disagree nor agree

4 = I agree

5 = I strongly agree

99 = Not answered

**Client Satisfaction Questionnaire**

1. To what extend has the iCBT intervention met your needs in treating depressed patients?

1 = None of my needs have been met

2 = Only a few of my needs have been met

3 = Most of my needs have been met

4 = Almost all of my needs have been met

99 = Not answered

2. In an overall general sense, how satisfied are you with the iCBT treatment you have provided?

1 = Quite dissatisfied

2 = Indifferently or mildly dissatisfied

3 = Mostly satisfied

4 = Very satisfied

99 = Not answered

3. If you were to provide treatment again, would you use the iCBT intervention again?

1 = No, definitely not

2 = No, I don´t think so

3 = Yes, I think so

4 = Yes, definitely

99 = Not answered

**Organisational Implementation Climate**

1. My supervisors are highly engaged in using the iCBT services in the clinical practice.

1 = I strongly disagree

2 = I disagree

3 = I neither disagree nor agree

4 = I agree

5 = I strongly agree

99 = Not answered

2. A visible and recognisable strategy is used in my organisation for implementing the services (Strategy that contains activities that are structured according to the following phases: planning, engaging, executing and evaluation).

1 = I strongly disagree

2 = I disagree

3 = I neither disagree nor agree

4 = I agree

5 = I strongly agree

99 = Not answered

3. Within the organisation, enough qualified staff is available to provide the services.

1 = I strongly disagree

2 = I disagree

3 = I neither disagree nor agree

4 = I agree

5 = I strongly agree

99 = Not answered

4. An adequate number of resources are made available to offer the services.

1 = I strongly disagree

2 = I disagree

3 = I neither disagree nor agree

4 = I agree

5 = I strongly agree

99 = Not answered

5. I am committed to the goals of the organisation I work for. Commitment in this can be interpreted in terms the extent to which individuals’ needs and expectations about the organisation are matched by their actual experiences.

1 = I strongly disagree

2 = I disagree

3 = I neither disagree nor agree

4 = I agree

5 = I strongly agree

99 = Not answered

6. I see myself as being loyal to the organisation I work for. Loyalty refers to the extent to which personnel are shares the goals, values and mission of the organisation. Loyal employees often feel the necessity to be with the organisation, in good and in bad conditions, and perform their job in an accurate and ordered manner.

1 = I strongly disagree

2 = I disagree

3 = I neither disagree nor agree

4 = I agree

5 = I strongly agree

99 = Not answered

7. I feel confident in my ability to use the iCBT services in treating clients.

1 = I strongly disagree

2 = I disagree

3 = I neither disagree nor agree

4 = I agree

5 = I strongly agree

99 = Not answered

8. I feel supported by my organisation in applying and using new ways of working such as iCBT in my daily practice.

1 = I strongly disagree

2 = I disagree

3 = I neither disagree nor agree

4 = I agree

5 = I strongly agree

99 = Not answered

9. I feel recognised by the organisation I work for when incorporating a new intervention in my daily practice.

1 = I strongly disagree

2 = I disagree

3 = I neither disagree nor agree

4 = I agree

5 = I strongly agree

99 = Not answered

10. I feel appreciated by the organisation I work for when incorporating a new intervention in my daily practice.

1 = I strongly disagree

2 = I disagree

3 = I neither disagree nor agree

4 = I agree

5 = I strongly agree

99 = Not answered

11. I am enthusiastic about using iCBT in treating clients.

1 = I strongly disagree

2 = I disagree

3 = I neither disagree nor agree

4 = I agree

5 = I strongly agree

99 = Not answered

12. I give high priority to implementing the iCBT services in my daily work.

1 = I strongly disagree

2 = I disagree

3 = I neither disagree nor agree

4 = I agree

5 = I strongly agree

99 = Not answered
